# Microrobots for Precision Diagnosis and Treatment in the Digestive System: A Review of Actuation Mechanisms, Structural Design, Preclinical and Translational Applications

**DOI:** 10.3390/mi17080973

**Published:** 2026-08-18

**Authors:** Yulong Gao, Fei Liu, Gongxin Li

**Affiliations:** The Key Laboratory of Advanced Process Control for Light Industry (Ministry of Education), Institute of Automation, Jiangnan University, Wuxi 214122, China

**Keywords:** microbots, digestive system, precision medicine, targeted delivery, gastrointestinal tract

## Abstract

Because lesions associated with digestive system diseases are often deeply seated, embedded within complex luminal milieus, and protected by substantial local delivery barriers, conventional diagnostic and therapeutic modalities remain constrained in their targeting capability, minimal invasiveness, and precision. Microrobots, leveraging micro-scale motion, active navigation, programmable controllability, and theranostic integration, open a new avenue for the precise diagnosis and treatment of digestive system diseases. Here, we review the major actuation modalities and structural designs of microrobots and discuss recent advances in their use across the gastrointestinal and hepatopancreatobiliary systems, focusing on targeted drug delivery, biospecimen harvesting, lesion detection, and interventional treatment. We further discuss the major obstacles to progress in this field, including robust operation in complex in vivo settings, real-time imaging and closed-loop feedback control, biosafety, degradability, and eventual clinical implementation. With continued advances in high-performance materials, multimodal actuation, intelligent control, and convergence with endoscopic and medical imaging technologies, microrobots are poised to accelerate the transition of digestive disease care toward precision, intelligence, and minimally invasive intervention.

## 1. Introduction

Digestive diseases constitute a substantial global public health burden, and colorectal cancer now ranks among the most commonly diagnosed malignancies worldwide, with its mortality burden increasingly shifting toward low- and middle-income countries. With more than one million new cases each year, gastric cancer remains a leading cause of mortality from gastrointestinal cancers [1,2]. Meanwhile, the incidence of inflammatory bowel disease continues to rise in newly industrialized countries, while functional gastrointestinal disorders, gastroesophageal reflux disease, and chronic hepatobiliary and pancreatic diseases markedly impair quality of life and intensify the socioeconomic burden [3]. Although current therapies, including systemic drug delivery, endoscopic interventions, radiotherapy, and surgery, can extend survival, they are broadly constrained by inadequate local drug exposure, substantial systemic toxicity, fluctuating efficacy, and the requirement for repeated invasive procedures [4,5,6]. In particular, the long, tortuous gastrointestinal tract and the anatomically complex biliary and pancreatic ducts continue to impede precise targeting, durable retention, and sustained therapeutic intervention [7,8]. Concurrently, marked heterogeneity in luminal diameter, geometry, mucosal organization, and pH across the gastrointestinal tract, together with gastric acid, digestive enzymes, mucus barriers, epithelial tight junctions, ongoing peristalsis, and fluid flushing, constrains stable transport and local retention of therapeutics and carriers, making it difficult for conventional passive delivery systems to reconcile stability, mucoadhesion, and spatiotemporal control [9,10,11,12,13]. These considerations underscore the urgent need for new therapeutic platforms capable of active navigation within the digestive system, long-term residence at target sites, and on-demand drug release or minimally invasive intervention.

Among emerging technological avenues, microrobots offer a new route to addressing the aforementioned challenges by virtue of their small size, high controllability, and programmable functionality [14,15,16,17]. A growing body of research over the past two decades indicates that microrobots that transduce exogenous or endogenous energy into directional locomotion can realize active actuation, controlled navigation, and precise delivery of drug payloads in complex biological fluids [18,19]. Particularly in the gastrointestinal tract, magnesium- or zinc-based chemical micromotors have provided in vitro proof of concept for disease treatment, as bubble thrust generated by the reaction between gastric acid and the metal enables these microrobots to penetrate the gastric mucus layer, actively adhere to the stomach wall and markedly improve drug utilization at the gastric mucosa [9,10,20]. In addition, enzyme-driven microrobots powered by urease, glucose oxidase, and related biocatalysts, together with externally actuated microrobots, have been used for oral and local drug delivery, in vivo imaging, and tissue repair in the digestive system. Distinct microrobot geometries also exhibit their unique performance characteristics; for instance, helical structures are more favorable for locomotion in narrow tubular environments, whereas spherical structures show superior advantages in steering.

To clarify the terminology and scope of this review, we distinguish five classes of miniature biomedical devices according to their characteristic dimensions, mobility, and access mode. Nanorobots or nanomotors refer to actively propelled or externally navigated systems whose functional mobile units are predominantly at the submicrometer scale, providing advantages in mucus or tissue penetration, local catalytic activity, and cellular-scale delivery [21]. Microrobots are defined here as controllably mobile systems with characteristic dimensions of approximately 1 μm to less than 1 mm, which are particularly suited to localized drug transport, micro-scale sampling, and minimally invasive manipulation [22,23]. Millirobots generally span approximately 1–10 mm and offer greater payload capacity and mechanical output for adhesion, biopsy, cargo transport, and tissue interaction [24,25]. Capsule robots are swallowable, encapsulated devices at the millimeter-to-centimeter scale; their larger internal volume permits the integration of cameras, batteries, communication units, sensors, sampling modules, and drug release mechanisms, although their gastrointestinal transit may be either passive or actively controlled [26]. Robotic catheters and continuum robots are tethered, elongated, and flexible instruments whose distal motion or shape is robotically actuated, providing high retrievability, payload capacity, and intervention force for endoluminal, biliary, or pancreatic procedures [27]. By contrast, passive smart carriers and conventional manually steerable instruments lack active locomotion, robotic actuation, or controllable reconfiguration and are therefore not treated as core microrobotic systems in this review.

Because size-based terminology is not fully standardized across miniature medical robotics, these categories are used as operational rather than regulatory definitions in this review. Device size alone is not sufficient for inclusion: the core evidence set comprises systems that provide active locomotion or steering, controlled shape reconfiguration, or remotely commanded diagnostic or therapeutic task execution [25]. Autonomy is not mandatory, and externally controlled, self-propelled, environmentally responsive, and partially autonomous systems are all eligible, provided that their mode of control is explicitly identified. Both untethered and tethered platforms are included, but functional ingestible capsules and robotic catheters or continuum robots are analyzed as separate translational classes because they differ substantially in access route, power source, clinical workflow, retrievability, manufacturing constraints, and regulatory pathway [28]. Passive nanoparticles, stimuli-responsive carriers without controllable mobility, and conventional guidewires or catheters without robotic actuation are excluded from the core robot category and are discussed only as comparators or enabling components.

Previous reviews have provided valuable classifications of micro-/nanorobots according to propulsion mechanisms, constituent materials, structural configurations, or drug delivery functions [14,29]. Notably, Wang et al. also discussed miniature robots in relation to gastrointestinal microenvironments, imaging modalities, and therapeutic applications [30]. At a broader translational level, Bozuyuk et al. proposed a field-wide roadmap for mobile medical microrobotics, emphasizing realistic validation in complex biological environments, application-specific microfabrication, biosafety and material stability, clinically compatible imaging, and close collaboration between microrobotics researchers and clinicians [31]. Therefore, the novelty of the present review does not lie merely in reorganizing the literature by anatomical site. Instead, we establish an organ-centered, clinically oriented framework that uses the anatomical and physiological constraints of each digestive organ as the starting point and links them to prospective clinical tasks, access routes, device scale, actuation strategy, structural and material requirements, functional outcomes, and translational barriers. This framework complements mechanism- or structure-based taxonomies by addressing a different question: which microrobotic design principles are most relevant to a specific digestive organ and prospective clinical scenario, and why?

Within this framework, we first summarize actuation mechanisms and structural configurations as cross-cutting engineering tools rather than independent endpoints of classification. We then compare how these tools must be adapted to the acidic and highly motile stomach, the long, mucus-rich and spatially heterogeneous intestine, and the narrow, difficult-to-access hepatobiliary and pancreatic ducts. By integrating targeted therapy with fluid or tissue sampling, lesion detection, sensing, and intraductal intervention, the review aims to translate fragmented engineering advances into organ-specific design principles and clinically relevant selection criteria.

## 2. Actuation Mechanisms of Microrobots

Microrobots exhibit diverse actuation mechanisms, and the mode of actuation is one of the core determinants of their locomotion performance, functional implementation, and application scope. To meet the demand for multimodal locomotion of microrobots within the complex physiological milieu of the digestive system, researchers have continuously pursued the development and refinement of different actuation mechanisms. At present, microrobot actuation is not limited to physical field actuation using magnetic, acoustic, and light field stimuli but also encompasses chemical and biological driving mechanisms, as shown in Table 1. This section evaluates actuation strategies according to the anatomical environment, physiological medium, access route, and intended task within the digestive system. Particular attention is given to whether a mechanism can provide sufficient propulsion or steering in acidic gastric fluid, non-Newtonian intestinal mucus, highly dynamic luminal environments, or confined biliary and pancreatic ducts.

### 2.1. Physical Field-Based Actuation

#### 2.1.1. Magnetic Field Actuation

**Figure 1:**
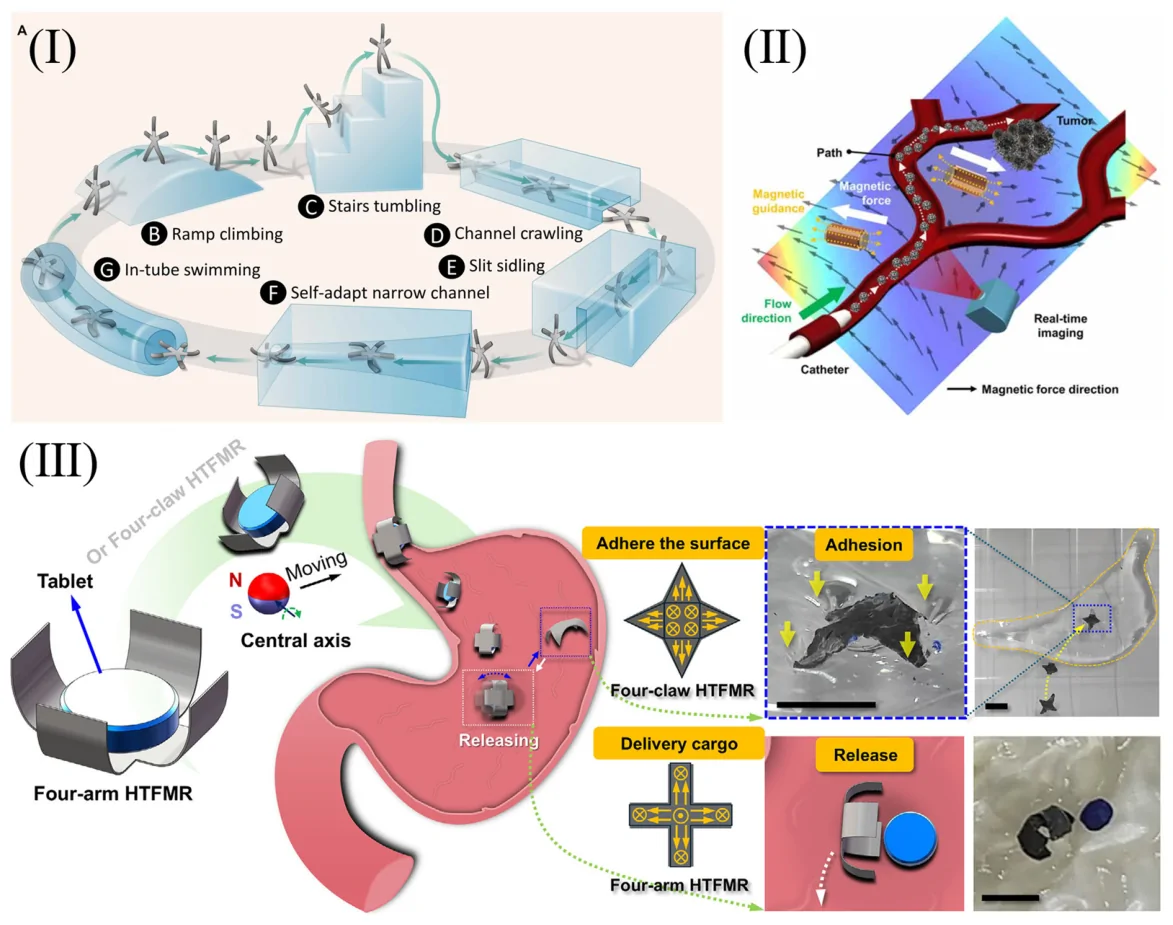
Magnetically actuated microrobots: (**I**) Magnetic microrobots exhibit multimodal locomotion in a complex obstacle-laden environment. Reproduced from Ref. [24] under the terms of the Creative Commons Attribution-NonCommercial 4.0 International License (CC BY-NC 4.0). (**II**) Workflow of in vivo tumor therapy enabled by a microrobotic system. Reproduced from Ref. [36] under the terms of the Creative Commons Attribution-NonCommercial 4.0 International License (CC BY-NC 4.0). (**III**) Schematic illustration of the reprogrammable, shape-retaining, flexible control, and cargo delivery and release functionalities of the HTFMR. Reproduced with permission from Ref. [38]. Copyright 2025 Wiley-VCH GmbH.

Magnetic field actuation is achieved by modulating externally applied magnetic fields, which induce magnetic forces and torques on microrobots, thereby enabling their precise motion. Owing to its high tissue penetration capability and flexible controllability [32], magnetic actuation has been widely employed for in vivo therapeutic and diagnostic applications [33]. Magnetic actuation is particularly suitable for active capsule navigation and sensing in the stomach, lesion targeting and mucosal retention in the intestine, and image-guided steering of catheter-compatible devices in the biliary and pancreatic ducts. Inspired by the morphology of the stag beetle, Chen et al. developed an untethered bioinspired soft robotic vehicle that can be precisely controlled by a rotating magnetic field generated by Helmholtz coils, enabling stable locomotion across diverse surfaces together with obstacle avoidance, path planning, and sensing capabilities (in vitro validation) [34]. By integrating immune cells with magnetic nanoparticles, Zheng et al. developed an ultrasoft hydrogel-based immunomillirobot capable of multimodal locomotion, including walking and tumbling, under magnetic field control (Figure 1I) (small-animal validation) [24]. Lee et al. designed a trilayer intestinal therapeutic agent delivery microrobot (ITAM) comprising a therapeutic core, a magnetic-nanoparticle targeting layer, and a protective outer layer, which, by combining magnetic targeting with a colon pH-triggered degradation mechanism, withstood the gastric environment after oral administration and precisely targeted colonic lesions in mice, thereby markedly suppressing colorectal cancer cells (small-animal validation) [35]. Go et al. developed a multifunctional microrobot composed of a porous architecture and surface-modified Fe_3_O_4_ nanoparticles, which, under magnetic guidance from a coil-based electromagnetic actuation module, achieved targeted vascular embolization and precise drug delivery in an orthotopic rat liver cancer model, resulting in marked tumor shrinkage and high targeting efficiency (Figure 1II) (small-animal validation) [36]. Jeon et al. fabricated porous magnetically controlled microrobots by 3D laser lithography and deposited a nickel–titanium layer, enabling rolling or helical locomotion under rotating magnetic fields, successfully supporting the culture and differentiation of hippocampal neural stem cells and allowing precise stem cell delivery in vitro, ex vivo, and in vivo (small-animal validation) [37]. Hu et al. designed a trilayer hydrogel-film magnetic millirobot that exhibits programmable deformation and shape retention under an external magnetic field, with a four-arm configuration for cargo capture and release and a four-claw configuration for mucosal adhesion, while maintaining excellent biocompatibility (Figure 1III) (in vitro validation) [38]. Magnetic field-actuated microrobots offer the advantages of non-invasive operation, good biocompatibility, and the ability to penetrate biological tissues for precise control. Meanwhile, magnetic torque and gradient force depend on the robot magnetic moment, applied field and field gradient, whereas the reachable workspace, control accuracy, and system-level power consumption depend strongly on field-generator geometry, working distance, actuation mode, and clinical workspace. Miniaturization generally reduces the available magnetic volume, but rolling, gradient pulling, rotating-field propulsion, surface-contact mechanics, or robot-arm-mounted magnetic sources can partly compensate for the reduced magnetic moment.

#### 2.1.2. Acoustic Actuation

Acoustic actuation offers characteristic advantages, including deep penetration, high flexibility, and good biocompatibility, making it highly attractive for many emerging applications, such as targeted drug delivery, particle separation, pumping, microsurgery, microfluidic manipulation, and chemical analysis. Liu et al. employed two-photon polymerization to print a microrobot comprising three groups of microtubes with distinct lengths, where acoustic bubble actuation together with a non-uniform mass distribution conferred autonomous self-righting, and frequency-selective activation of individual microtubes produced vertical translation and clockwise or counterclockwise yaw in a 3D space (in vitro validation) [39]. Inspired by the ciliary architecture of starfish larvae, Dillinger et al. harnessed micro-scale nonlinear acoustic effects to induce small-amplitude oscillations of artificial cilia, giving rise to source-like bulk fluid motion as well as microrobot actuation and microparticle capture (in vitro validation) [40]. This design circumvented the limitations of the Scallop theorem to enable micro-scale acoustic actuation and, through the integration of adjacent positive and negative ciliary bands, achieved microparticle capture and transport that mimicked the feeding mechanism of starfish larvae. Kaynak et al. established an integrated framework for the design, manufacture, and control of 3D-printed soft robotic microsystems, in which ultrasound transduction enabled frequency-selective actuation and supported the development of multiple wireless biomanipulation tools, including multi-pump composite micromachines, thus validating their potential for minimally invasive diagnostics and targeted treatment (in vitro validation) [41]. Hao et al. introduced a focused ultrasound-controlled phase transition (FUPT) strategy that exploited the acoustothermal effect of iron oxide nanoparticles to induce the phase transition of low-boiling-point liquids, thereby achieving millimeter-scale spatially selective actuation and Newton-scale force output for untethered soft robots. The same strategy was subsequently used to develop a pipeline liquid-delivery robot and an intestinal biopsy-repair robot (in vitro validation) [25]. Han et al. used two-photon polymerization to fabricate bioabsorbable acoustic microrobots (BAMs) featuring optimized surface chemistry and a dual-opening bubble-trapping cavity design, which enabled stable acoustic actuation under focused ultrasound stimulation, efficient locomotion in multiple biological fluids, and hydrolysis-mediated biodegradation (small-animal validation) [23]. Aghakhani et al. 3D-printed hollow acoustic microrobots with oscillating microbubbles, in which a chamber design incorporating doubly concave microstructures enhanced bubble stability, while acoustic actuation generated high-shear-rate fluid flow, giving rise to a surface-slipping actuation mode in Newtonian fluids and a puller-type actuation mode in non-Newtonian fluids, thereby providing a new strategy for microrobot deployment in minimally invasive targeted therapy (in vitro validation) [42]. Acoustic actuation is most applicable to fluid-filled gastric and intestinal environments when localized propulsion, mucus penetration, or remotely triggered mechanical output is required. High-shear acoustic microstreaming can support locomotion through viscoelastic mucus, whereas focused ultrasound can provide spatially selective actuation for intestinal manipulation or repair; however, performance depends strongly on acoustic pressure, frequency, cavity geometry, and biofluid rheology.

#### 2.1.3. Light Actuation

Light-driven microrobots convert optical energy into propulsive forces or shape morphing motions via photothermal, photochemical, or photomechanical mechanisms, allowing precise control [43]. Because direct optical actuation requires sufficient light penetration and optical access, it is most defensible for endoscopically accessible regions of the stomach or colon and for localized activation of robots that have already reached the target site. Palagi et al. used structured monochromatic light to actuate microrobots composed of photosensitive liquid-crystal elastomers, enabling selectively addressable functionalities based on traveling-wave locomotion. The robots achieved autonomous actuation in the absence of external force or torque, mimicked synchronous or antisynchronous ciliary beating modes, and displayed diverse gaits and versatile modes of locomotion (in vitro validation) [44]. Qin et al. integrated plasmonic nanomotors and customized plasmonic nanotweezers into microrobots and used an unfocused circularly polarized laser beam to achieve nanometer-scale trajectory control while stably trapping nanodiamonds, thereby executing complex operations including capture, transport, release and recapture (in vitro validation) [45]. Li et al. developed a 3D-printed, three-tubular rocket-like multinozzle microrobot whose gold coating enhanced optical absorption and photoacoustic signals and, by coupling near-infrared actuation with optical-resolution photoacoustic microscopy, enabled high-speed locomotion in a blood-mimicking viscous medium together with precise tracking in blood at 3.2 μm resolution (in vitro validation) [46]. Zhang et al. adopted a truncated-octahedral lattice architecture and achieved sequential actuation by modulating laser scanning frequency, path, and power, yielding a light-driven lattice soft microrobot capable of linear crawling, in situ rotation, and jumping (in vitro validation) [47]. Deng et al. fabricated a ring-shaped structure with a zero-elastic-energy mode from photothermally responsive liquid-crystal elastomers (LCEs); under constant illumination, the structure generated a thermal gradient through the photothermal effect, underwent spontaneous rotation, and exploited dynamic friction or viscous drag to realize light-controlled locomotion across solid surfaces, confined spaces, and fluidic environments (in vitro validation) [48]. By directly activating resting macrophages with a focused near-infrared beam and optically steering their extended pseudopodia for directional navigation, Li et al. constructed a light-controlled microrobot that precisely phagocytosed nanoplastics, microorganisms, and cancer-cell debris both in vitro and in live zebrafish (small-animal validation) [49]. Although light-driven systems feature high energy density and fast response, their shallow penetration depth, reliance on external high-intensity illumination, susceptibility to environmental interference, and the limited mechanical performance of certain materials largely confine their applications to superficial tissues.

#### 2.1.4. Electric Field Actuation

In electric field actuation, applied fields induce ionic migration, dipolar alignment, or electrostatic interactions within the soft materials of the robot, converting electrical energy into mechanical deformation that drives microrobotic locomotion and function. On the basis of concentration-polarization electro-osmosis, Katzmeier et al. introduced a concentration-polarization electrophoretic actuation mechanism, in which joystick-controlled modulation of the direction and frequency of a planar alternating electric field enabled arbitrary two-dimensional locomotion together with programmable cargo capture, transport, release, and particle-chain assembly (in vitro validation) [50]. Wu et al. used self-propelled metal-dielectric Janus particles as mobile microelectrodes and, under an external electric field, exploited dielectrophoresis-induced local electric field enhancement together with continuous alternating fields or alternating pulse signals to achieve the selective capture, transport, and electroporation of bacteria such as *Escherichia coli* (in vitro validation) [51]. Ying et al. developed an electroadhesive hydrogel interface (e-GLUE) integrating cationic polymers with a tough hydrogel matrix and demonstrated that electrical stimulation promotes active polycation diffusion into deep mucosal layers and cross-linking with polyanionic proteins, resulting in robust retention on porcine gastrointestinal mucosa (large-animal validation) [52]. Electrical actuation is most suitable for endoscopically accessible gastric or intestinal mucosal surfaces, where locally delivered electrodes can provide temporary fixation, drug delivery, hemostasis, or biosensing. It is less suitable for long-range free-swimming propulsion or deep biliary and pancreatic navigation because its effective range and safety depend on tissue conductivity, electrode configuration, stimulation duration, and electrochemical by-products.

### 2.2. Chemical Actuation

#### 2.2.1. Hydrogen Peroxide Actuation

Hydrogen peroxide-based actuation converts chemical energy into kinetic energy, with the gas evolved during H_2_O_2_ reactions driving microrobot motion. By using the electrochemical decomposition of hydrogen peroxide, Sánchez et al. achieved autonomous actuation and developed robots integrating autonomous locomotion, sensing, cargo transport, and actuation, which could be wirelessly controlled by fuel gradients and related cues (in vitro validation) [53]. By confining catalytically active platinum nanoparticles within the nanocavities of polymeric stomatocytes, Wilson et al. generated thrust via catalytic H_2_O_2_ decomposition, enabling autonomous and directional motion (in vitro validation) [54]. Villa et al. used low concentrations of hydrogen peroxide as fuel to fabricate tubular TiO_2_ microrobots decorated with platinum nanoparticles via atomic layer deposition and photodeposition, yielding a system with antibacterial activity, biocompatibility, and the capacity to activate immune responses against microbial infection (in vitro validation) [55]. Guo et al. developed a one-end-open yolk-shell microrobot that underwent diffusiophoretic actuation through H_2_O_2_ gradients established by single-atom Fenton chemistry and photocatalytic redox reactions, releasing multiple reactive oxygen species during motion and ultimately achieving in vitro killing of *Escherichia coli* (in vitro validation) [56]. Using a water-in-oil double-emulsion solvent-evaporation strategy, Yang et al. developed a H_2_O_2_-powered PLGA-Pt micromotor whose gas-driven actuation actively traversed the colonic mucus barrier, thereby confirming strong mucopenetrating performance and enabling effective therapy for colorectal cancer (small-animal validation) [57]. Reaction-driven chemical propulsion is strongly organ dependent: gastric acid-powered metal microrockets are suitable for penetrating gastric mucus and sustained intragastric delivery, whereas effervescent or hydrogen peroxide-responsive systems are more suitable for intestinal mucosal penetration or inflamed colonic environments. These approaches are less appropriate for biliary or pancreatic intervention because fuel availability, reaction rate, by-product safety, and retrieval are difficult to control.

#### 2.2.2. Enzyme Actuation

**Figure 2:**
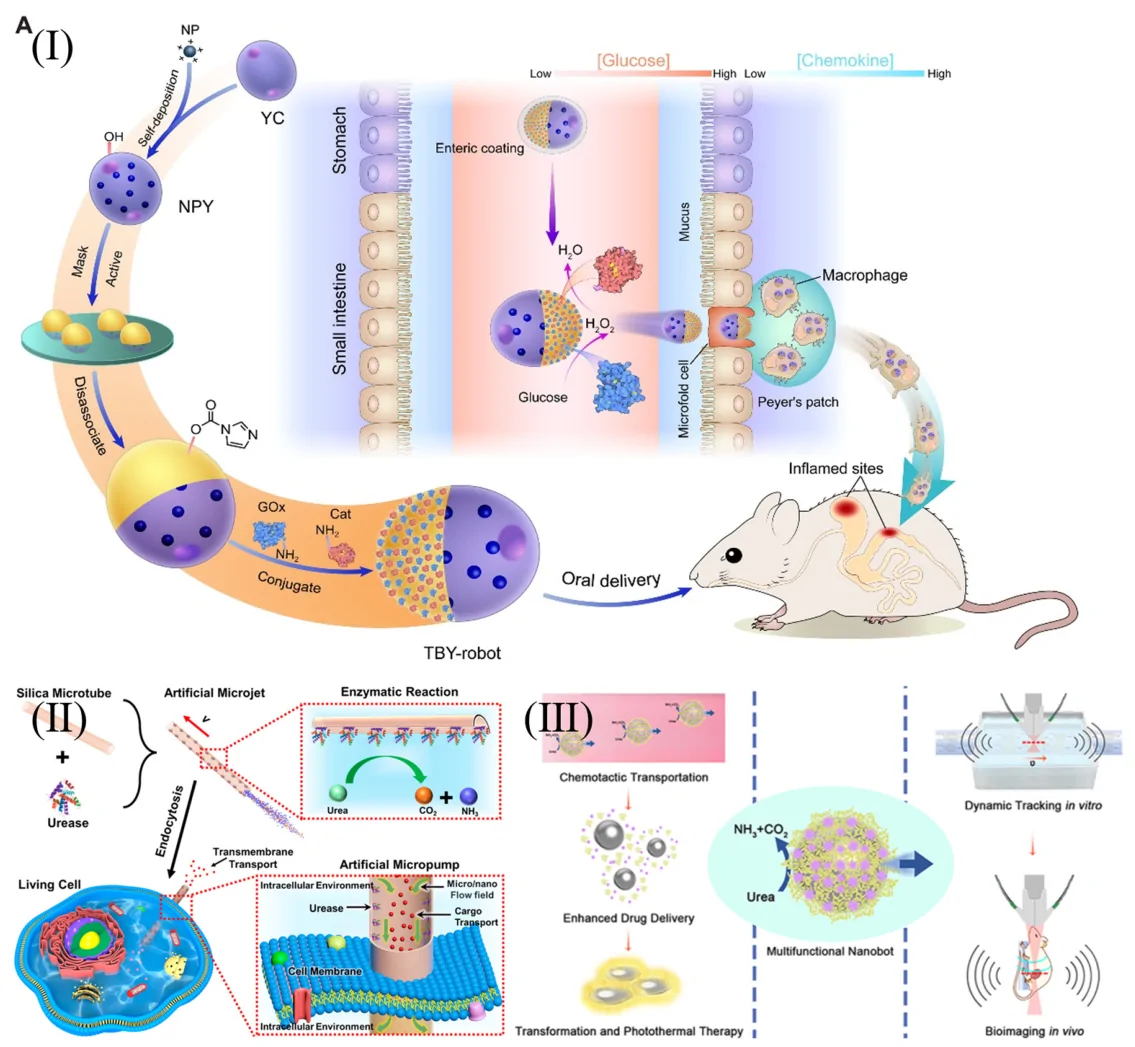
Enzyme-driven microrobots: (**I**) Twin-bioengine yeast microrobots achieve autonomous navigation within the gastrointestinal tract. Reproduced from Ref. [58] under the terms of the Creative Commons Attribution-NonCommercial 4.0 International License (CC BY-NC 4.0). (**II**) Enzyme-powered tubular microjets enable active transmembrane cargo transport. Reprinted with permission from Ref. [59]. Copyright 2023 American Chemical Society. (**III**) An enzyme-powered (urease-powered) liquid metal (LM) nanobot with multiple biomedical functionalities. Reprinted with permission from Ref. [61]. Copyright 2021 American Chemical Society.

Compared with the cytotoxicity associated with hydrogen peroxide, actuation strategies based on the enzymatic catalysis of fuels such as urea and glucose are considered safer. Depending on enzyme distribution, robot geometry, substrate gradients, and interfacial properties, enzymatic reactions can generate propulsion through bubble recoil, ionic or neutral diffusiophoresis, chemophoresis, enzyme-induced microflows, or chemotactic migration along substrate gradients. By engineering a dual-bioengine yeast micro-/nanorobot, Zhang et al. realized adaptive actuation and targeted delivery through enzyme–macrophage switching (Figure 2I) (small-animal validation) [58]. Using a template-assisted chemical synthesis approach, Wang et al. anchored urease onto silicon-based microtubes to produce an enzyme-powered tubular microrobot that achieved self-actuation through microflows generated by urea decomposition and created artificial transmembrane microchannels in living cells, thereby improving the transmembrane transport efficiency of anticancer agents (Figure 2II) (in vitro validation) [59]. Tang et al. asymmetrically immobilized urease on the surface of natural platelets to fabricate Janus platelet microrobots, which used urea as a biofuel for autonomous actuation while preserving the intrinsic targeting capability of platelets and enhancing binding efficiency to cancer cells and bacteria (in vitro validation) [60]. Xu et al. constructed a urease-powered nanorobot composed of polydopamine-coated biocompatible liquid metal that could undergo chemotactic transport along urea gradients (Figure 2III) (small-animal validation) [61]. Enzyme actuation offers the advantage of high autonomy but remains limited by insufficient propulsive power. For digestive applications, the suitability of enzyme propulsion therefore depends on local substrate availability, enzyme stability across gastric and intestinal pH conditions, resistance to proteolytic degradation, and the persistence of physiologically meaningful chemical gradients.

### 2.3. Biological Actuation

#### 2.3.1. Cell Actuation

**Figure 3:**
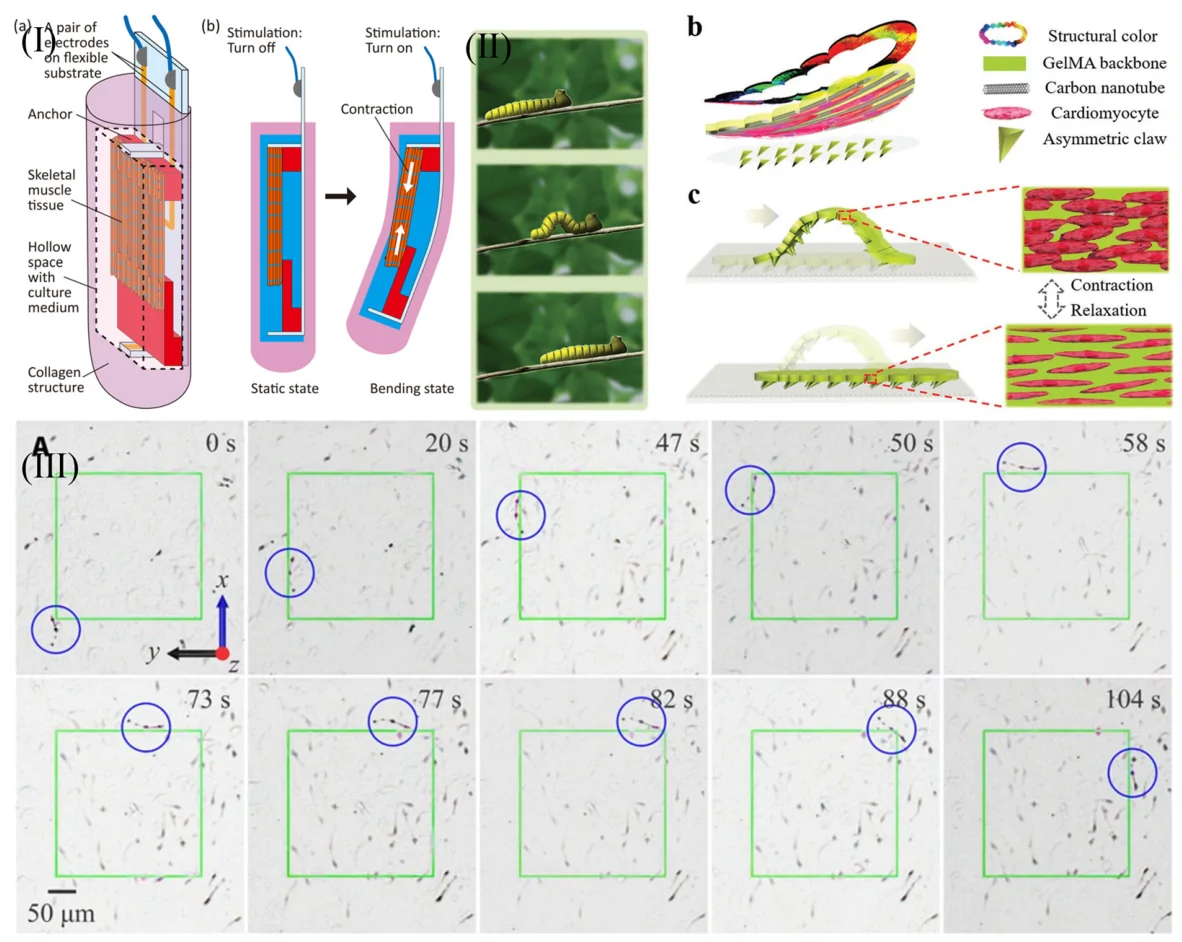
Cell-driven microrobots: (**I**) Concept of a biohybrid robot with a skeletal muscle tissue encapsulated with a collagen structure. Reproduced from Ref. [63] under the terms of the Creative Commons Attribution 4.0 International License (CC BY 4.0). (**II**) The crawling process of the soft robot driven by cardiomyocytes. Reproduced with permission from Ref. [65]. Copyright 2019 WILEY-VCH Verlag GmbH & Co. KGaA, Weinheim. (**III**) A biohybrid magnetic microrobot composed of a sperm cell and magnetic nanoparticles moving along a square trajectory near a boundary. Reproduced from Ref. [66] under the terms of the Creative Commons Attribution-NonCommercial 4.0 International License (CC BY-NC 4.0).

In cell-driven propulsion, microrobot locomotion is powered by the inherent motility and functional attributes of living cells, most notably cardiomyocytes, skeletal muscle cells, and sperm cells. Kinjo et al. reported a bipedal biohybrid robot actuated by cultured skeletal muscle tissue, in which electrically triggered muscle contraction enabled forward locomotion, halting and more precise steering than that of conventional counterparts (in vitro validation) [62]. Morimoto et al. integrated cultured skeletal muscle tissue with a collagen-supported robotic structure, demonstrating that engineered muscle contraction could provide sustained locomotor actuation (Figure 3I) (in vitro validation) [63]. Williams et al. fabricated PDMS microfilaments by capillary drawing, selectively seeded cardiomyocytes onto these filaments, and, guided by slender-body hydrodynamic modeling, engineered a self-propelled biohybrid microrobot operating at low Reynolds number regimes, thereby establishing a foundational platform for increasingly sophisticated biological machines (in vitro validation) [64]. Drawing inspiration from the crawling mechanisms of snakes and caterpillars, Sun et al. engineered a microrobot comprising asymmetric claws, a CNT-induced cardiomyocyte tissue layer, and a structural-color reporting layer, a configuration that enabled directional crawling. This microrobot can be incorporated into a multi-track microfluidic chip system to move along predefined tracks, with its crawling speed modulated by the local stimulus concentration, thus highlighting its potential for clinical disease-screening applications (Figure 3II) (in vitro validation) [65]. Magdanz et al. combined electrostatic self-assembly between immotile sperm cells and magnetic nanoparticles with the regularized Stokeslet model to fabricate a soft-magnetic microrobot endowed with ultrasound-based localization, drug-loading capability, and good biocompatibility (Figure 3III) (in vitro validation) [66]. Current evidence is insufficient to assign purely cell-driven microrobots to a specific digestive organ. Chemotactic macrophages may be useful as biological relay carriers for inflamed gastric or colonic tissues, but this function has so far been demonstrated mainly in hybrid enzyme–cell systems rather than independently cell-actuated gastrointestinal robots.

#### 2.3.2. Microbial Actuation

Compared with cells, microorganisms feature small size, low mass, and superior viability [78] and have thus also been extensively used to power microrobots. Microbial actuation strategies mainly fall into two categories: microalgae-driven and bacteria-driven. In microalgae-based platforms, directional locomotion is typically achieved through phototaxis and related tropic behaviors, whereas self-regulated motion is mediated by flagellar beating. Building on the phototactic behavior of microalgae, Xie et al. developed an algal guidance system in which dynamically modulated light cues directed microalgal locomotion, thereby enabling reciprocating swimming in microfluidic chips, traffic-rule-compliant traversal of intersections, and movement along arbitrarily prescribed trajectories, including zigzag and triangular paths (in vitro validation) [67]. Ha et al. fabricated an algal biohybrid microrobot by electrostatically assembling drug-loaded, pH-responsive peptide nanotubes (PNTs) onto the surface of *Chlamydomonas*; this design preserved agile locomotion while enabling phototactic targeting of cancer cells and pH-triggered drug release (small-animal validation) [68].

Bacteria-driven propulsion harnesses the intrinsic onboard motility and sensing machinery of bacteria to actuate bacteriabots. Park et al. fabricated bacterial microrobots by selectively patterning *Salmonella Typhimurium* on the surface of polystyrene microbeads and found that these selectively patterned bacteriabots exhibited substantially higher motility than counterparts uniformly covered with bacteria (in vitro validation) [69]. Using Escherichia coli Nissle 1917 (EcN) as the biological engine, Wang et al. developed a pH-responsive EcN-driven microrobot that specifically targets hypoxic regions of intestinal tumors, undergoes bacterium–particle separation under acidic conditions, and is subsequently internalized by tumor cells, thereby establishing a safe and feasible strategy for targeted therapy of hypoxic intestinal tumors (small-animal validation) [70]. Buss et al. engineered a biohybrid microswimmer by integrating nanoerythrosomes with genetically engineered Escherichia coli, which outperformed several comparable designs in motility while simultaneously reducing hydrodynamic drag (in vitro validation) [71]. Microbial actuation is most suitable for the intestine and colon, where probiotic bacteria or microalgae can exploit hypoxia, inflammation, pH, or chemical gradients for tumor or inflammatory-site targeting. Gastric applications require enteric protection because acidity and digestive enzymes may impair microbial viability, while biosafety, proliferation control, and host–microbiome interactions remain major constraints.

### 2.4. Hybrid Actuation

Hybrid actuation encompasses a broad range of strategies, including multiphysics actuation, physical–chemical actuation and physical–biological actuation. A central advantage of multiphysics-driven microrobots lies in the complementary integration of distinct field effects, enabling improved control precision, faster dynamic response, richer functionality, and thus a wider application scope. Peng et al. realized non-contact transport of micro-scale cargos via a coupled hybrid actuation strategy combining negative phototaxis under optical stimulation and precise guidance under rotating magnetic fields (in vitro validation) [72]. Gao et al. fabricated a magnetically actuated photoelectric hybrid microrobot that exhibited axial rolling, radial rolling, and swinging under rotating magnetic fields and, upon visible-light illumination, generated a photoelectric response enabling precise targeting and activation of neuronal cells (in vitro validation) [73]. de la Asunción-Nadal et al. reported a biohybrid microrobot driven by both light and magnetic fields, enabling precise motion control over a full 360° range (in vitro validation) [74]. Cherukumilli et al. engineered a bioinspired bipedal microwalker based on magneto-acoustic hybrid actuation, in which magnetic fields afforded precise manipulation at low speed whereas acoustic fields supplied rapid propulsion, thus enabling transport of hamster ovarian cells and patterning of colloidal particles (in vitro validation) [75]. Although multiphysics hybrid actuation enables complex manipulation and multifunctional integration, its practical implementation faces increasingly sophisticated coordinated-control design, stricter material and fabrication constraints, more demanding control and safety validation, and greater challenges in modeling and robustness caused by multi-field coupling, as well as persistent limitations in biocompatibility, energy efficiency, and clinical translation.

Physical–chemical and physical–biological hybrid actuation schemes combine the strengths of distinct actuation mechanisms, thus expanding the flexibility of microrobotic applications. Celik Cogal et al. realized hybrid actuation by combining chemical energy from hydrogen peroxide catalysis with acoustic energy generated by surface acoustic waves, thereby boosting locomotion speed and enabling precise motion control via the acoustic field [76]. Zhang et al. developed a biohybrid microrobot that, when actuated by an external rotating magnetic field, could actively traverse the mucus barrier, extend residence time, and retain favorable biocompatibility (small-animal validation) [77].

Recent gastrointestinal robots further demonstrate that actuation should be co-designed with local rheology, surface interactions, field delivery, and the intended clinical task. High-shear acoustic propulsion enabled locomotion through non-Newtonian mucus [42], whereas magnetically guided low-friction robots supported locomotion in complex gastrointestinal environments, mechanical biofilm disruption under low-frequency fields, and magnetocaloric bacterial eradication under high-frequency fields [79]. Low-friction magnetic capsules and ciliated microcatheters further enabled controlled navigation and sensing in the porcine intestine and image-guided biliary intervention, respectively [27,80]. Hybrid actuation is particularly suitable for multibarrier intestinal and colonic applications in which protected gastrointestinal transit, mucus penetration, lesion targeting, and local therapy cannot be achieved by a single propulsion mechanism. Sequential enzyme–macrophage propulsion and bacteria–ultrasound combinations illustrate how different actuation stages can be matched to different physiological barriers, although control complexity and safety requirements increase accordingly. Owing to pronounced mismatches in spatiotemporal scales and modes of action among different actuation mechanisms, these systems remain simultaneously constrained in complex physiological environments by insufficient biocompatibility, attenuated propulsion efficiency, coupled motion control, and difficulties in manufacturing standardization and clinical translation, making it difficult to simultaneously achieve efficient actuation, precise controllability, long-term stability, and practical applicability.

## 3. Structural Configurations of Microrobots

Structural design is a critical factor governing the overall performance and functional capabilities of microrobots, and suitably designed soft microrobots hold considerable promise for applications in the gastrointestinal system. Rational structural design can enhance the motility of microrobots while simultaneously meeting the stringent demands of biocompatibility and safety. At present, structural design strategies are based not only on geometric configurations and morphological features, but also on the coupled selection of functional materials, fabrication methods, magnetization patterns, surface properties, and physical programming strategies. These factors collectively determine whether a microrobot can deform, adapt to its surroundings, adhere to or detach from biological surfaces, release its payload, or switch between different locomotion modes. Collectively, these diverse structural designs have expanded the application landscape of microrobots and laid a solid foundation for their deployment in the gastrointestinal system, as shown in Table 2.

### 3.1. Helical Structures

**Figure 4:**
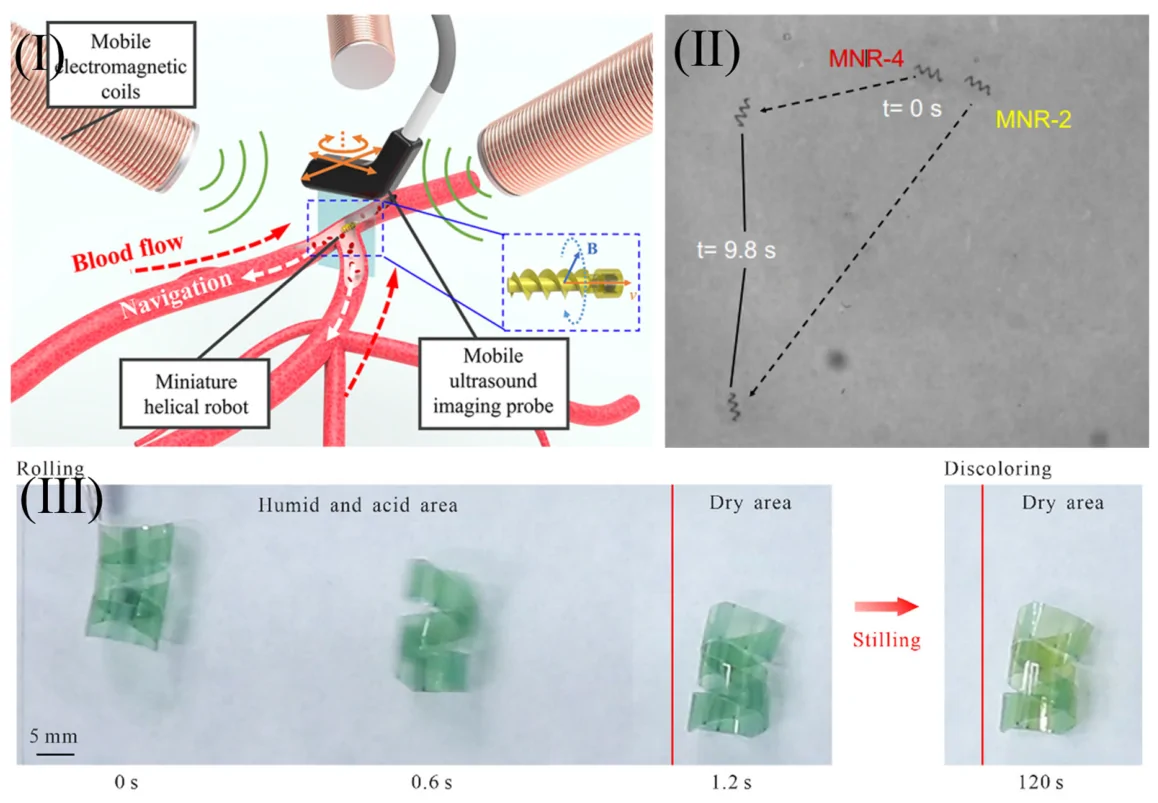
Helical microrobots: (**I**) An ultrasound Doppler imaging-guided helical microrobot for targeted thrombolysis. Reprinted with permission from Ref. [81]. Copyright 2022 American Chemical Society. (**II**) A comprehensive fluid–solid interaction dynamic model that enables precise navigation for general helical micro-/nanorobots. Reproduced from Ref. [82] under the terms of the Creative Commons Attribution-NonCommercial-NoDerivatives 4.0 International License (CC BY-NC-ND 4.0). (**III**) A double-helical soft robot driven by constant humidity that achieves ultra-fast rolling locomotion through alternating deformation of two helices. Reproduced from Ref. [83] under the terms of the Creative Commons Attribution 4.0 International License (CC BY 4.0).

The gastrointestinal tract predominantly presents low Reynolds number liquid environments, in which viscous drag dominates micrometer-scale locomotion, rendering reciprocal actuation ineffective for net propulsion. Accordingly, helical microrobots inspired by flagellated bacteria have been widely adopted for use within the gastrointestinal tract. Wang et al. reported autonomous navigation of helical microrobots in dynamic blood flow by integrating a hybrid magnetic torque–force control strategy with Doppler ultrasound guidance (Figure 4I) (in vitro validation) [81]. Hu et al. established a dynamic model based on the fluid–structure interaction theory and, using standard and generalized helical microrobots fabricated via two-photon polymerization, showed that the model accurately predicts the propulsion speed, transient dynamics, and surface stress distributions of various such robots (Figure 4II) (in vitro validation) [82]. Dreyfus et al. developed a magnetic continuum robot with helical ribs and a hinged magnetic head, achieving rotation and linear translation through the combination of a triangular electromagnetic navigation system and a motor-driven propulsion module (large-animal validation) [28]. Xu et al. engineered a double-helical closed-loop robot from a humidity-responsive agarose film, enabling ultra-fast rolling in a constant humidity environment and the transport of payloads equivalent to its self-weight (Figure 4III) (in vitro validation) [83]. Zhao et al. developed an optically controlled helical microrobot by transforming manually stretched and twisted strips into a 3D helical configuration, thereby achieving directional rolling, such as leftward turning, rightward turning, and straight-line motion, together with high robustness and reversible shape morphing (in vitro validation) [84]. Fan et al. fabricated a cross-scale ferrofluidic helical microrobot by sculpting helical features into a PMMA substrate with a bent needle tip, enabling targeted drug delivery under complex physiological conditions (ex vivo validation) [85]. Zhu et al. designed a magnetic helical microrobot incorporating a soft-magnetic manipulation gripper, enabling reliable cargo transport in glycerol under low Reynolds number conditions (in vitro validation) [86]. Helical architectures enable microrobots to generate effective propulsion in low Reynolds number fluids and navigate confined tubular environments with comparatively strong thrust; however, they remain constrained by limited maneuverability and the risk of damaging biological tissues. Meanwhile, helical pitch, magnetic anisotropy, surface roughness, and material stiffness jointly regulate propulsion efficiency, contact-induced friction, and tissue interaction. These parameters are particularly important when adapting helical robots from relatively simple fluidic models to mucus-rich or anatomically confined digestive environments.

### 3.2. Spherical Structures

Spherical microrobots are typically constructed from Janus particles, while designs integrating microorganisms with magnetic materials have also been developed to enable locomotion and additional functionalities. In addition, the structural simplicity of spherical microrobots facilitates scalable fabrication and supports swarm control and multirobot applications. Ning et al. employed Pickering-type double emulsions as templates to successfully fabricate tunable Janus microspheres exhibiting dual anisotropy in porosity and magnetism, thereby enabling oriented alignment under magnetic fields (in vitro validation) [87]. Li et al. fabricated magnetically actuated microrobots with a burr-like porous spherical architecture by means of 3D laser lithography, thereby achieving targeted cell loading and delivery (small-animal validation) [88]. Wei et al. developed biodegradable microrobots featuring a spiky porous spherical architecture, which enabled the precise delivery of engineered stem cells through gradient magnetic actuation combined with photoacoustic imaging guidance (small-animal validation) [89]. In addition to magnetic actuation, spherical microrobots can also be propelled chemically. Sun et al. reported Janus nanocatalytic spherical robots capable of hydrogen peroxide-driven self-propulsion, which, together with real-time MRI guidance, facilitated deeper tumor penetration (small-animal validation) [90]. Luo et al. prepared, via microfluidics, sodium alginate microspheres incorporating M2 macrophage membrane-coated Janus nanomotors, whereby the alginate matrix conferred tolerance to the gastric environment and controlled release, and MnO_2_-mediated catalysis of H_2_O_2_ produced O_2_ to drive the motors across the mucus barrier (small-animal validation) [91]. For spherical and Janus microrobots, anisotropic surface chemistry, porosity, and magnetic or catalytic domain distribution are often more important than the external spherical geometry itself. These features determine cargo loading, catalytic propulsion, mucus penetration, and stimulus-responsive release, thereby linking spherical architecture to specific gastrointestinal tasks. Owing to their structural simplicity, efficient functional integration, ease of large-scale fabrication, and suitability for swarm applications, spherical microrobots are attracting growing attention for medical applications, especially in the gastrointestinal tract.

### 3.3. Multilegged Structures

**Figure 5:**
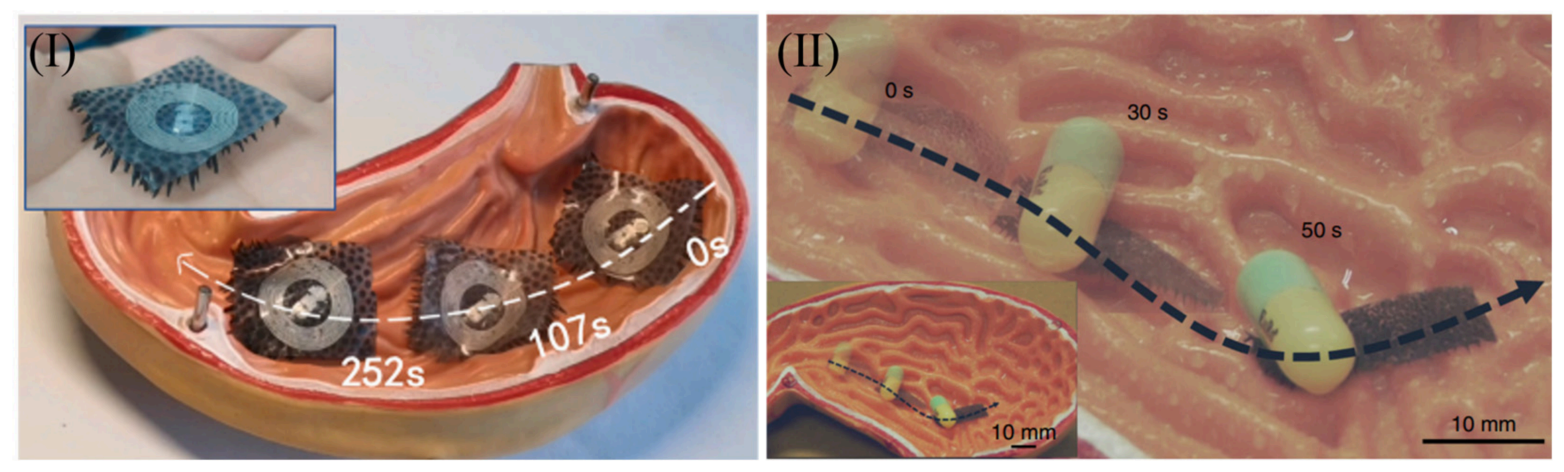
Multilegged microrobots: (**I**) A liquid metal multilegged robot capable of multidirectional and multimodal locomotion for targeted delivery. Reproduced from Ref. [95] under the terms of the Creative Commons Attribution-NonCommercial-NoDerivatives 4.0 International License (CC BY-NC-ND 4.0). (**II**) A multilegged microrobot equipped with flexible conical feet, enabling locomotion across wet and slippery substrates. Reproduced from Ref. [97] under the terms of the Creative Commons Attribution 4.0 International License (CC BY 4.0).

Multilegged microrobots draw inspiration primarily from millipedes and centipedes, whose multilegged morphologies facilitate efficient traversal of complex terrain via rhythmic, wave-like leg kinematics [92,93]. Such a crawling gait further confers enhanced stability over uneven substrates. The multilegged crawling mode supports robust locomotion in complex environments such as the gastrointestinal tract. Drawing inspiration from millipede morphology, Wang et al. engineered a sawtooth body coupled with an array of magnetic legs and fabricated the multilegged robot via a two-step mold-casting strategy, enabling rhythmic-wave propulsion under external magnetic actuation [94] (in vitro validation). Likewise inspired by millipedes, Ye et al. integrated elastomers with magnetic nanoparticles to construct multilayer legs and developed a liquid metal-enabled, flexible-electronics-integrated multilegged robot capable of curled rolling, surfacing diving, and crawling, with functionality in acidic environments for gastrointestinal-targeted delivery (Figure 5I) (in vitro validation) [95]. Inspired by centipedes, Hoffman et al. developed a kinematics-guided, multisegment microrobot whose modular architecture permits segment addition, removal, and replacement of damaged components, thereby supporting swarm and multilegged robotic control (in vitro validation) [96]. Inspired by biological pedal architectures, Lu et al. developed an untethered, multilegged soft millirobot featuring multiconical soft feet, which exhibited versatile locomotion under external magnetic actuation and robust performance in both dry and wet settings, highlighting its potential for intragastrointestinal drug transport (Figure 5II) (in vitro validation) [97]. Inspired by starfish, Yang et al. constructed a pentaradially symmetric robot with biomimetic tube feet from a PDMS–iron particle composite, wherein the tube-foot architecture reduced locomotor friction and improved obstacle-negotiation capability, thereby conferring strong environmental adaptability (in vitro validation) [98]. Gu et al. reported a bilayer multilegged millirobot capable of magnetically guided operation and precise drug release in acidic media, demonstrating promise for targeted gastrointestinal drug delivery (in vitro validation) [99]. By recapitulating the biomechanical principles of natural multilegged crawlers, multilegged microrobots can attain high adaptability, improved energetic efficiency, and multifunctional performance. The adaptability of multilegged robots arises from the interaction between leg geometry, elastomeric compliance, magnetic programming, and surface friction. Soft feet, deformable legs, and independently programmed magnetic segments can improve traction, reduce slipping, and facilitate locomotion across wet, uneven, or acidic gastrointestinal surfaces.

### 3.4. Other Structures

Owing to the complexity of the intraluminal gastrointestinal environment, site-specific tasks require microrobots with different structural designs, among which tubular, star-shaped, rocket-like, guidewire-like, and modular architectures are commonly adopted in addition to the configurations described above. Different microrobot geometries exhibit distinct characteristics and environmental adaptability, allowing each design to fulfill specific functions under different operational challenges [105,106]. Tubular microrobots feature a simple architecture and are particularly advantageous for cargo transport, drug release, and body fluid collection. Yan et al. developed anisotropically magnetized tubular microrobots that could switch between rolling and tumbling modes through frequency modulation of an external rotating magnetic field. The robot could adapt to complex environments and exploit local microvortices generated during locomotion-mode switching to achieve microfluidic manipulation, thereby enabling the precise capture, transport and release of microparticles (in vitro validation) [100]. Wang et al. generated metastable tubular microrobots by rolling superparamagnetic colloidal spheres in a precessing magnetic field, enabling precise magnetic regulation of propulsion direction and speed (in vitro validation) [101]. Hu et al. prepared star-shaped hydrogel microrobots by casting a mixed hydrogel polymer into a star-shaped mold, and these devices achieved efficient, controllable propulsion under a vertically rotating magnetic field together with good biodegradability and mechanical robustness (in vitro validation) [102]. Rocket-shaped microrobots combine structural stability with strong propulsion, which makes them well suited for gastrointestinal applications such as drug delivery and sensing [46]. Wan et al. reported a metal–organic framework-based microrocket fabricated by template-assisted electrodeposition, in which self-propulsion was driven by hydrogen bubbles generated from the reaction of zinc with hydrochloric acid in gastric fluid; following penetration of the gastric mucus barrier, the device achieved controlled and sustained drug release (small-animal validation) [103]. Zhang et al. developed a three-dimensional magnetic guidewire system that enabled bidirectional 180° deflection in 3D space by correlating guidewire bending behavior with the strength and orientation of the applied magnetic field (in vitro validation) [104]. Modular microrobots have also shown outstanding performance because the reversible combination and separation of multiple modules improve system functionality and provide an important technical foundation for operation in the complex, dynamic gastrointestinal tract [34]. Wang et al. developed a modular magnetically actuated capsule robot that combined covalently crosslinked hydrogel surface modification with multimodal locomotion to achieve continuous gastrointestinal and temperature monitoring, thus integrating propulsion, control and sensing into a single platform (ex vivo validation) [80]. Meanwhile, the work of Sitti and colleagues illustrates how fabrication strategy and material composition can be directly linked to robotic function. For example, machining-inspired fabrication enables ferrofluidic helical robots with multimodal locomotion, while wireless soft robots use morphology and magnetic response as indirect indicators of tissue adhesion, pH, and viscoelasticity [24,85,107]. With the growing demands of practical applications, a broad range of microrobot architectures has been devised to resolve or mitigate the challenges encountered in this field. Structural designs of gastrointestinal microrobots will continue to be refined and optimized to satisfy the demands of real-world operation and device engineering.

## 4. Applications of Microrobots in the Digestive System

Compared with conventional therapeutic modalities, microrobots hold considerable promise for applications in the digestive system; however, their practical deployment remains highly challenging owing to the dynamic intraluminal milieu, tortuous anatomical pathways, mucus-rich interfaces, and complex pH landscapes. At present, microrobotic applications in the digestive system mainly target intraluminal or intra-organ interventions in the stomach, intestines, liver, biliary tract, and pancreas, covering navigation and positioning, drug delivery and controlled release, biosensing, and targeted therapy and thus providing strong support for the detection, diagnosis, and treatment of gastrointestinal diseases.

### 4.1. Stomach

#### 4.1.1. Intragastric Targeted Drug Delivery

As an essential organ of the digestive system, the stomach imposes significant constraints on microrobot deployment owing to its distinctive and multifaceted physiological milieu. The gastric mucosa constitutes the primary protective and functional barrier of the stomach, characterized by a highly folded surface yet limited absorptive area, resulting in a drug residence time generally shorter than three hours [108]. The highly corrosive and dynamic nature of gastric fluids exerts a selective pressure on exogenous materials, necessitating that microrobots or drug carriers entering the stomach possess robust stability to prevent structural degradation or premature drug leakage. With a pH maintained between 1 and 4 [109], the stomach provides a highly acidic milieu where most drugs undergo oxidation, deamidation, or hydrolytic degradation, leading to reduced bioactivity [30,110]. Moreover, the abundance of digestive enzymes in the stomach exhibits potent degradative activity, which can compromise natural polymer-based materials, necessitating that microrobotic carriers exhibit resistance to enzymatic degradation or employ protective mechanisms for drug encapsulation [111]. Collectively, these structural and physicochemical characteristics of the stomach impose stringent requirements on microrobotic systems, prompting extensive efforts in their design and application.

In gastric-targeted drug delivery, microrobots commonly rely on external remote actuation or self-propulsion driven by gastric acid or enzymatic reactions to achieve active navigation and precise localization at specific gastric sites, with mucosal-adhesive coatings or specific binding moieties improving gastric retention. Upon reaching the target site, the microrobots rapidly release therapeutic payloads, thereby markedly increasing local drug concentration, enhancing therapeutic efficacy, and minimizing off-target toxicity to healthy tissues [112,113]. Chen et al. developed a trilayered magnetic soft microrobot whose tailored interlayer magnetic interactions, combined with external magnetic field actuation, enabled translational and rolling locomotion modes as well as on-demand disassembly, allowing precise navigation to ulcer sites and targeted adhesion (large-animal validation) [114]. Sun et al. designed a magnetically driven capsule in which the competitive interplay between magnetic gradient forces and magnetic torque enabled flexible valve switching and a multifrequency response control strategy decoupled global motion from local responses, facilitating targeted drug delivery, sampling, dual-drug selective release, mucus clearance, and photothermal-assisted therapy (small-animal validation) [26]. Liu et al. developed a magnetic capsule robot capable of withstanding esophageal pressure to prevent premature drug leakage while enabling active intragastric locomotion and on-demand precise drug release (in vitro validation) [115]. Darmawan et al. fabricated a bilayer pH-responsive microrobot that folds under alkaline conditions and unfolds in acidic environments, enabling precise drug-mediated eradication of gastric cancer cells (ex vivo validation) [116]. Ávila et al. developed magnesium-based microrobots capable of active drug delivery and significantly reducing gastric bacterial load, with a chitosan coating enhancing adhesion and retention to the gastric wall, thereby enabling safe and effective treatment of gastric infections (small-animal validation) [117]. Inspired by octopus suction cups, Tong et al. designed OISR microrobots in which the suction structures function as drug reservoirs, enabling precise navigation to gastric lesion sites, followed by localized sustained drug release (in vitro validation) [118]. Despite extensive applications of microrobots in targeted intragastric drug delivery, limitations in real-time in vivo imaging and closed-loop navigation, insufficient evidence regarding biosafety and biodegradability, and high barriers to manufacturing standardization and clinical translation further constrain their reliability, reproducibility, and clinical feasibility in realistic gastric environments.

#### 4.1.2. Gastric Fluid Sampling

Gastric fluid sampling is achieved by microrobots navigating to and residing at target regions within the stomach, where transient opening of the sampling inlet is triggered via magnetic valves [119], structural deformation [120], or material-triggered mechanisms, allowing gastric fluid to be drawn into the chamber through negative pressure or absorbent materials, followed by valve reset, swelling-induced sealing, or dedicated sealing structures to isolate the sample and prevent contamination from subsequent gastrointestinal contents. Wu et al. developed a multicompartment magnetic capsule robot based on compliant mechanisms and programmable magnetic actuation, in which gradient magnetic fields achieve selective opening of individual chambers while rotating magnetic fields induce rolling locomotion, thereby enabling targeted fluid sampling in the gastrointestinal tract (ex vivo validation) [121]. Mandsberg et al. designed a metal-free, battery-free ingestible gastric fluid sampling device that enables non-invasive sampling of gastric fluid while also addressing scalability in manufacturing and shelf-life stability (large-animal validation) [122]. Although microrobotic gastric fluid sampling provides a novel approach for non-invasive, in situ acquisition of gastric biochemical and microbiological information, it remains challenged by limited understanding of gastric physiology, the harsh acidic and dynamically evolving digestive environment, and inter-individual variability in gastric motility that complicates precise spatiotemporal localization, together with unresolved issues in sealing and structural reliability after chamber actuation, which collectively preclude the simultaneous achievement of sample representativeness, integrity, and reproducibility.

#### 4.1.3. Intragastric Detection

Microrobots equipped with onboard microcameras and wireless communication modules can achieve active steering, precise localization and systematic scanning of the gastric lumen under external magnetic actuation, addressing the long-standing challenge of incomplete mucosal coverage due to the large gastric volume [123,124]. Upgraded imaging sensing capabilities improve the visualization of mucosal folds and minute lesions, enabling enhanced imaging-based identification and localization of pathologies including inflammation, ulceration, hemorrhage and early-stage malignancies [125].

**Figure 6:**
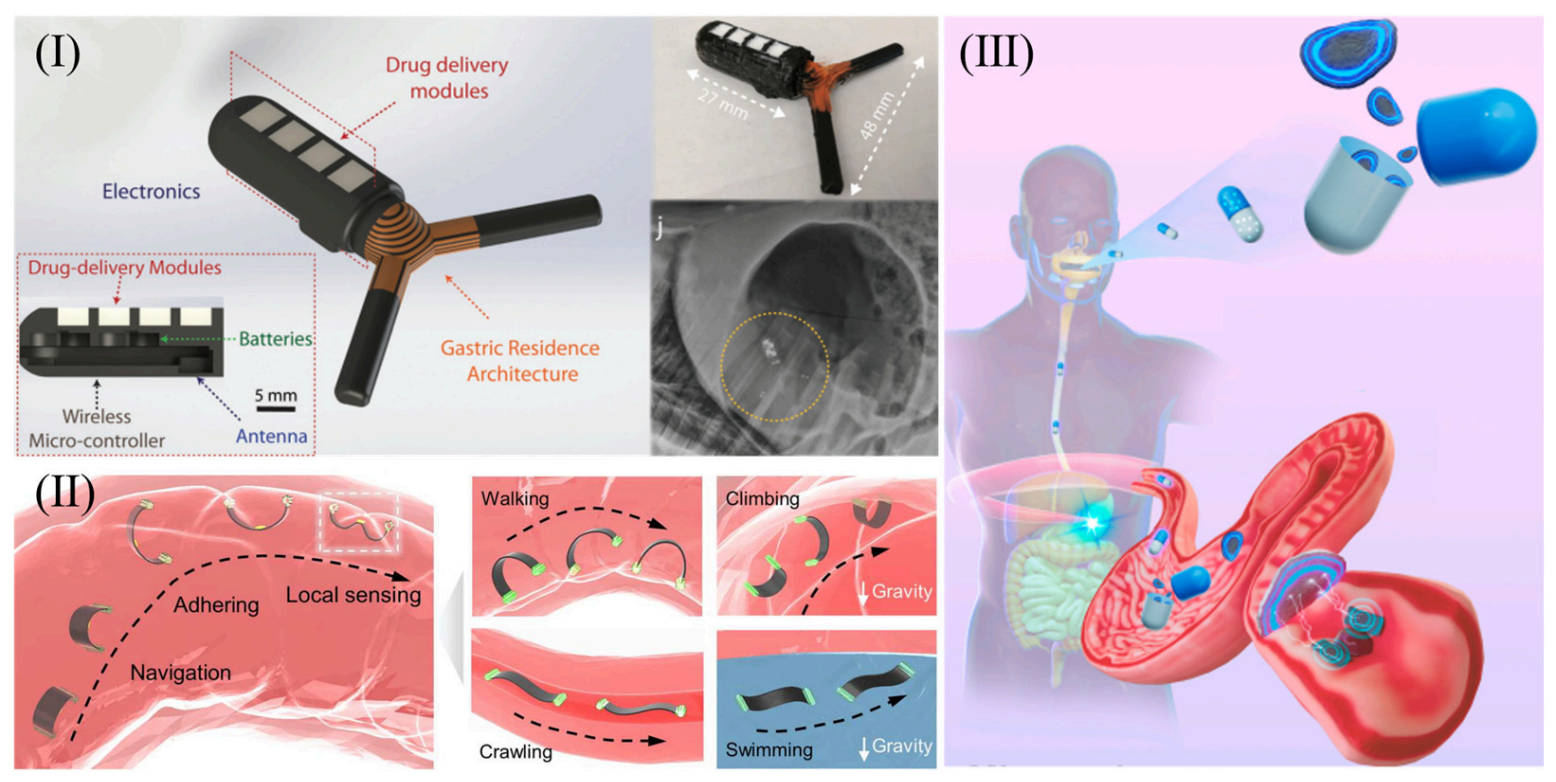
Intragastric applications of microrobots: (**I**) A wireless gastric-resident electronic device capable of long-term retention and remote communication within the stomach. Reproduced from Ref. [129] under the terms of the Creative Commons Attribution 4.0 International License (CC BY 4.0). (**II**) A wirelessly actuated miniature soft robot for in situ sensing of physiological properties of soft tissues. Reproduced from Ref. [107] under the terms of the Creative Commons Attribution 4.0 International License (CC BY 4.0). (**III**) Soft robots with Cy5 for intraoperative navigation of gastric lesion. Reproduced from Ref. [131] under the terms of the Creative Commons Attribution 4.0 International License (CC BY 4.0).

Onboard sensing includes image acquisition, electrophysiological recording, biochemical detection, and physiological parameter monitoring. Recent capsule-endoscopy studies have further incorporated multi-task neural networks to recognize multiple lesion-related features from magnetically controlled capsule images, supporting computer-assisted gastric diagnosis [126]. Mimee et al. developed an ingestible microbial-electronic device in which engineered probiotics sense biomarkers and emit luminescent signals that are wirelessly transmitted to external receivers, enabling precise diagnosis of gastrointestinal bleeding (large-animal validation) [127]. You et al. developed an ingestible electrogastrography device capable of wirelessly transmitting biopotential signals, thereby enabling high-fidelity recording of gastric signals and monitoring motility changes induced by prokinetic agents (large-animal validation) [128]. Kong et al. developed a gastric-resident electronic platform featuring a reconfigurable structure that enables post-ingestion expansion and retention in the stomach, achieving long-term communication in a porcine gastric model via a Bluetooth wireless module (Figure 6I) (large-animal validation) [129]. Embodied or morphology-based sensing infers tissue or environmental properties from robot deformation, magnetic response, locomotion resistance, and contact behavior. Wang et al. demonstrated that wireless soft robots could infer tissue adhesion, pH, and viscoelasticity from robot morphology and magnetic field inputs, establishing a conceptual bridge between structural deformation and physiological sensing (Figure 6II) (ex vivo validation) [107]. Artificial cilia sensing provides an additional route for active fluid–structure interaction. Han et al. developed magnetically actuated artificial cilia integrated with flexible strain sensors and machine learning-based signal interpretation, enabling the recognition of fluid apparent viscosity, environmental boundaries, and flow speed (in vitro validation) [130]. This approach is particularly relevant to gastrointestinal environments in which mucus rheology, wall proximity, and local flow conditions directly influence microrobot locomotion and retention. External localization and imaging provide information about robot position, orientation, mucosal coverage, lesion location, and tissue response. He et al. used Cy5-labeled soft robots to localize gastric lesions around implanted magnetic clips in a rat model (Figure 6III) (small-animal validation) [131]. Chen et al. further integrated a retractable camera, magnetic navigation, and microneedle-mediated delivery in a robotic capsule platform, demonstrating an integrated diagnostic–therapeutic workflow in ex vivo porcine intestinal tissue (ex vivo validation) [132]. State estimation and control determine whether sensing information can be converted into reliable robotic behavior. Turan et al. developed a particle-filter-based multisensor fusion framework that combines magnetic localization with visual odometry to estimate capsule pose and identify sensor failures in an ex vivo porcine stomach model (ex vivo validation) [133].

Thus, a diagnostic microrobot should be evaluated not only by the accuracy of individual measurements but also by whether the measurements improve localization, path planning, disturbance rejection, or task execution. Despite these advances, gastrointestinal microrobotic diagnosis remains limited by the lack of standardized sensing metrics, incomplete calibration under realistic mucus and peristaltic conditions, uncertain correspondence between robot-derived signals and clinical diagnostic endpoints, and insufficient integration of sensing, localization, and control into a validated closed-loop workflow. Most reported systems remain at the in vitro, ex vivo, or animal validation stage and therefore should not yet be considered established clinical diagnostic platforms.

### 4.2. Intestine

#### 4.2.1. Intestinal Targeted Drug Delivery

The intestine is anatomically divided into the small intestine and the large intestine, with the former comprising the duodenum, jejunum, and ileum and the latter consisting of the cecum, colon, and rectum; the small intestine measures approximately 6 m in length, whereas the large intestine is ~1.5 m, making it the most complex and longest component of the digestive system [134]. The small intestinal mucosa is characterized by extensive folding and a high density of villous structures, which markedly increase the absorptive surface area and permit prolonged retention of luminal contents for as long as 9 h. Intestinal pH undergoes pronounced spatial fluctuations, with values around 6 in the duodenum, rising to ~7.4 at the distal ileum, dropping below 6 in the cecum, and then increasing along the colon to approximately 6.7 in the rectum [135]. Moreover, the presence of diverse enzymes and dense microbial populations within the gut milieu imposes significant constraints on the stability and operational efficacy of microrobotic systems [136].

**Figure 7:**
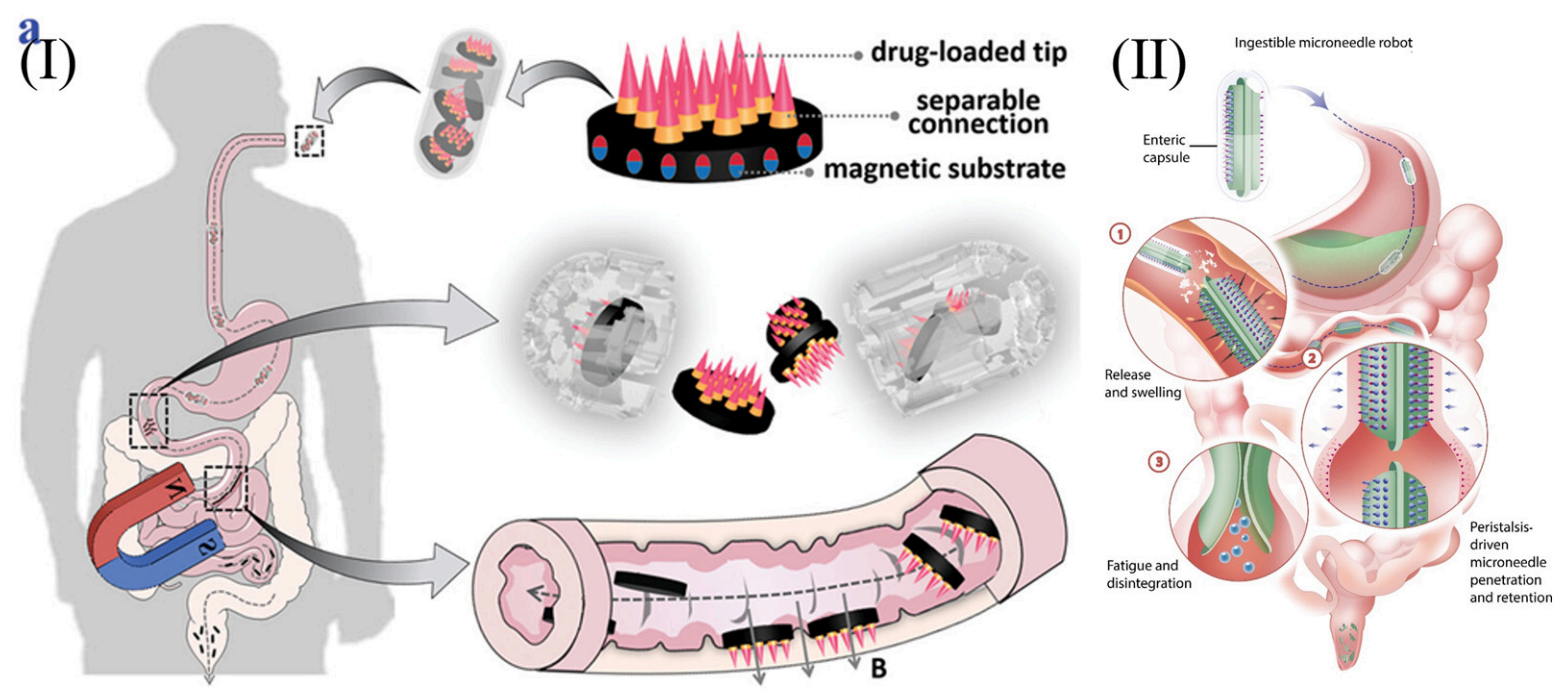
Microrobots for targeted intestinal drug delivery: (**I**) A magnetically responsive microneedle robot for intestinal drug delivery. Reproduced with permission from Ref. [141]. Copyright 2021 Wiley-VCH GmbH. (**II**) A peristaltically driven robot equipped with barbed microneedles for oral delivery of biopharmaceuticals. Reproduced from Ref. [144] under the terms of the Creative Commons Attribution 4.0 International License (CC BY 4.0).

Analogous to gastric-targeted drug delivery, microrobots can exploit diverse propulsion strategies, including enzyme-powered and magnetic actuation, to actively penetrate the intestinal mucus barrier, navigate toward the intestinal epithelium, and subsequently achieve site-specific drug delivery to diseased regions via chemotactic gradients [137,138,139]. Wu et al. fabricated Mg-based microrobots integrated into enteric capsules, where near-infrared light induced capsule disassembly to release the microrobots, thereby facilitating deep tissue imaging and accurate manipulation in the intestine, with enhanced propulsion contributing to extended residence time (small-animal validation) [140]. Zhang et al. employed a LEGO-inspired multistage 3D fabrication strategy to construct magnetically responsive microneedle robots; following entry into the small intestine, the enteric capsule disintegrated to release the robots, which were then guided by an external magnetic field to orient and penetrate the intestinal wall, enabling sustained drug release via needle tips retained within the tissue (Figure 7I) (large-animal validation) [141]. Zhou et al. designed a pH-responsive self-disintegrating capsule, in which the reaction between sodium bicarbonate and citric acid generated CO_2_ bubbles to trigger structural disassembly and drug release, thereby enabling efficient and precise delivery (small-animal validation) [142]. Kang et al. constructed biohybrid microrobots that, following oral administration, were released from microcapsules in the intestine, effectively suppressing primary colorectal tumors as well as hepatic and pulmonary metastases (small-animal validation) [143]. Inspired by the defensive inflation behavior of porcupinefish, Gao et al. developed peristalsis-driven microneedle robots that rapidly swell upon intestinal fluid absorption, enabling peristaltic contractile forces to facilitate tissue penetration, followed by detachment and mucosal retention of the barbed microneedles for sustained drug delivery (Figure 7II) (large-animal validation) [144]. Although intestinal microrobots demonstrate notable potential in prolonging drug residence and enabling accurate site-specific delivery, their clinical translation is hindered by poor adaptability to the complex gut milieu, reliance on biocompatible fuels or bulky external actuation systems, limited endurance, and inadequate verification of long-term stability and reliability.

#### 4.2.2. Intestinal Tissue Sampling

Intestinal tissue sampling by microrobots is predominantly achieved via passive, active, or dynamic strategies, leveraging technologies such as pH-sensitive coatings, magnetic guidance, and shape-memory alloys to enable targeted and contamination-free sampling; nevertheless, current designs still face limitations in miniaturization, self-localization, sampling efficiency, and translational feasibility [145,146,147]. Nejati et al. developed an electronics-free smart capsule with a dual-layer pH-responsive enteric coating for targeted activation, wherein superabsorbent hydrogels collect gut microbiota and a mechanical seal preserves the sample, allowing precise, compositionally intact sampling of proximal colon microbiota (large-animal validation) [148]. Del-Rio-Ruiz et al. designed a 3D-printed flexible ingestible device in which dissolution of a pH-dependent enteric coating triggers sampling, while superabsorbent sodium polyacrylate beads actuate an elastic microvalve to autonomously lock and prevent contamination, enabling non-invasive collection of small intestinal contents (ex vivo validation) [149]. Park et al. developed a magnetically actuated active capsule equipped with a micropump and microfluidic channels, enabling controlled locomotion and fluid extraction via electromagnetic actuation for repeated, site-specific intestinal microbiota sampling with negligible cross-contamination (ex vivo validation) [150]. Rehan et al. developed an untethered, autonomous robotic capsule for intestinal microbiota sampling, actuated by bidirectional shape-memory alloy springs. Optimization of the battery, sealing materials, and capsule materials enabled the first microbial acquisition from both the intestinal lumen and mucosal layer (ex vivo validation) [151]. Huang et al. designed a dual-functional ingestible passive capsule integrating pH-responsive and mechanically driven dual-control mechanisms, in which optimization of the pH-sensitive membrane, superabsorbent polymer sampling module, and spring-based sealing system enabled high-throughput intestinal sampling and targeted drug delivery within a swallowable form factor (large-animal validation) [152]. Although microrobotic intestinal tissue sampling provides a novel minimally invasive strategy for biopsy in deep and tortuous intestinal segments, its practical application remains significantly constrained by difficulties in precise localization and stable anchorage within peristaltic and folded intestinal environments, coupled with constraints imposed on sampling mechanisms at the micro-scale.

### 4.3. Liver, Pancreas and Biliary System

As accessory organs of the digestive system, the liver, biliary system, and pancreas contribute critically to overall digestive function. The deployment of microrobots within these organs typically necessitates navigation through highly confined ductal environments—for instance, the bile duct (~1–2 mm in diameter [153]) and the pancreatic duct (~3 mm [154])—thereby imposing stringent constraints on device dimensions. Furthermore, both bile (pH ~7.4) and pancreatic fluid (pH ~7.8) constitute a weakly alkaline environment; coupled with the requirement that orally delivered microrobots transit the gastrointestinal tract, this underscores the need to ensure their physicochemical stability throughout gastrointestinal transit.

**Figure 8:**
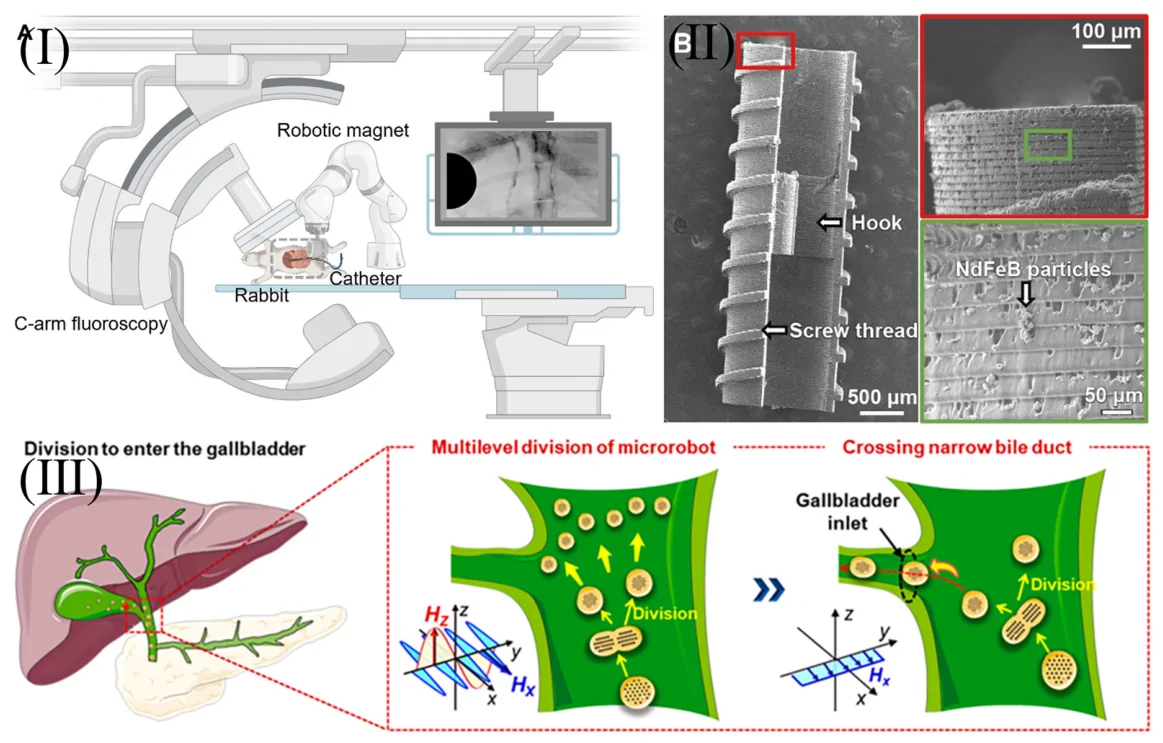
Microrobots for hepatobiliary and pancreatic applications: (**I**) A magnetically actuated liquid-infused sliding microcatheter designed for precise intrabiliary navigation. Reproduced from Ref. [27] under the terms of the Creative Commons Attribution-NonCommercial 4.0 International License (CC BY-NC 4.0). (**II**) A modular magnetic microrobotic system for minimally invasive treatment of stenotic bile ducts. Reproduced from Ref. [157] under the terms of the Creative Commons Attribution-NonCommercial 4.0 International License (CC BY-NC 4.0). (**III**) A cell-mimetic droplet microrobot that traverses the bile duct for intracholecystic drug release. Reproduced from Ref. [158] under the terms of the Creative Commons Attribution 4.0 International License (CC BY 4.0).

Microrobotic applications in the bile duct have demonstrated remarkable performance [155]; inspired by foxtail grass, Yang et al. engineered a magnetically actuated, cilia-integrated injectable sliding microcatheter, where oscillatory magnetic cilia improve maneuverability in tortuous pathways and minimize friction, enabling precise deployment of helical microswimmers into the bile duct (Figure 8I) (small-animal validation) [27]. Using 3D printing, Su et al. fabricated magnetically actuated modules that exhibit strong magnetic responsiveness and pH-responsive deformation, enabling on-demand disassembly in acidic environments under low-frequency rotating magnetic fields, thereby achieving targeted cell delivery and subsequent module retrieval in a rabbit bile duct model (small-animal validation) [156]. Building upon this, the team further designed a modular magnetically controlled microrobot integrating ultrasound-responsive self-expanding components, allowing real-time ultrasound-guided navigation and thermally triggered expansion, thereby achieving agile maneuvering in complex ductal phantoms and enabling minimally invasive intervention for biliary strictures (Figure 8II) (ex vivo validation) [157]. By employing alternating magnetic fields to program gas–liquid interface-encapsulated magnetic particle assemblies, Yu et al. generated cell-mimetic droplet microrobots capable of fission and exocytosis-like functionalities, enabling magnetically guided navigation to the bile duct and gallbladder for controlled drug release (Figure 8III) (in vitro validation) [158]. Liu et al. engineered biomimetic nanorobotic drug carriers cloaked with macrophage membranes to evade immune recognition and subsequent clearance, exhibiting enhanced bioavailability and extended circulation time and enabling site-specific drug release in the pancreas and regions with elevated reactive oxygen species (ROS) (small-animal validation) [159]. Calmé et al. developed a hybrid-actuated robotic platform by integrating tendon-actuated continuum robots with magnetically controlled soft continuum robots and validated its superior performance in precise positioning, reduced procedural trauma, and adaptability to diverse anatomical pathways during pancreatic interventions using a silicone pancreaticobiliary phantom (in vitro validation) [160]. Lloyd et al. enhanced the mechanical robustness of magnetically controlled soft continuum robots by embedding longitudinally braided nylon structures—and through rigid-link modeling, anisotropic material characterization, and genetic algorithm-based optimization of magnetization profiles—achieved tissue interaction-free 180° bending and autonomous pancreaticobiliary navigation within a planar ultrasoft pancreatic phantom (in vitro validation) [161]. Although microrobots hold promise for overcoming the limited accessibility of conventional instruments to deep-seated biliary and pancreatic duct strictures, they typically require catheter-mediated proximal deployment prior to precise navigation, introducing procedural complexity. These technical demands, together with the inherent risks of pancreatitis, cholangitis, hemorrhage, and perforation associated with pancreatobiliary endoscopic interventions, continue to hinder their clinical translation.

## 5. Conclusions and Future Directions

Currently, the management of gastrointestinal disorders predominantly depends on oral pharmacotherapy, endoscopic interventions, and surgical procedures, yet significant limitations remain in terms of precision, accessibility, and safety. Oral or systemic drug administration is susceptible to gastric acid, digestive enzymes, the mucus layer, and epithelial barriers, resulting in insufficient drug accumulation at target lesions and increased systemic side effects; while endoscopic and surgical interventions enable localized intervention, their inherent invasiveness limits sustained and precise therapy for deep-seated, complex, or diffuse lesions. Accordingly, there is a critical need to develop advanced technologies that enable active navigation, targeted delivery, and on-demand therapeutic interventions within the highly complex gastrointestinal milieu.

Microrobotic systems offer a new paradigm for gastrointestinal therapy, enabling a transition from passive drug delivery toward active, intervention-driven treatment. Recent progress has led to the development of microrobots actuated by magnetic, acoustic, chemical, and multimodal actuation, with magnetic actuation emerging as the most promising approach due to its excellent wireless controllability and deep tissue penetration. Structurally, diverse configurations—including helical, spherical, and multilegged designs—have been developed, enabling the integration of locomotion control, drug transport, mucus penetration, and stimuli-responsive release functionalities. In terms of applications, microrobotic systems have demonstrated promising potential in targeted drug delivery and sampling within the gastrointestinal tract, as well as in intraductal interventions in the biliary and pancreatic ducts. However, most digestive system microrobots remain at the in vitro, ex vivo, or small-animal stage, and technical functionality alone should not be interpreted as translational readiness. For digestive applications, these requirements should be assessed using organ- and task-specific benchmarks. Gastric systems require testing under acidic, mucus-rich, distensible, and peristaltic conditions; intestinal systems require non-Newtonian mucus, luminal flow, folded anatomy, and clearance to be reproduced; and hepatopancreatobiliary systems additionally require millimeter-scale duct models, catheter-compatible deployment, atraumatic navigation, retrievability, and failure recovery. Relevant quantitative endpoints include navigation success, localization error, speed and force at clinically realistic working distances, payload retention and release, residence time, tissue injury, retrieval success, and reproducibility relative to current endoscopic or catheter-based standards.

Furthermore, safety assessment should extend beyond short-term cytocompatibility to include chemical and mechanical stability, degradation-product biodistribution and clearance, local and systemic toxicity, immunogenicity, repeated exposure, sterilization compatibility, and contingency strategies for stalled or unretrieved devices. Scalable manufacturing additionally requires defined critical quality attributes, batch-to-batch consistency, sterilization, packaging, shelf stability, and standardized reporting of actuation conditions, robot dose, failure modes, and task-specific performance. Digestive microrobots should similarly progress from organ-relevant in vitro testing to ex vivo or anatomical-phantom validation, small-animal demonstration of biological efficacy, large-animal assessment of access and clinical workflow, and ultimately carefully designed first-in-human studies, with predefined go/no-go criteria and continuous feedback among engineers, clinicians, manufacturers, and regulatory specialists.

Looking ahead, this field is expected to evolve toward highly biocompatible materials, multimodal actuation, theranostic integration, and intelligent closed-loop control while further integrating with endoscopic technologies, medical imaging, and artificial intelligence to enhance operability and translational potential in real clinical settings.

## Figures and Tables

**Table 1:** Comparison of different actuation mechanisms of microrobots.

Actuation Mechanisms		Suitable Organs	Representative Tasks	References
Physical Field-based Actuation	Magnetic field	Stomach; intestine; hepatopancreatobiliary ducts.	Navigation; localization; delivery; sampling; intraductal intervention.	[24,32,33,34,35,36,37,38]
	Acoustic	Stomach; intestine; mucus-rich interfaces.	Mucus penetration; localized propulsion; delivery; tissue manipulation.	[23,25,39,40,41,42]
	Light	Endoscopically accessible stomach and colon.	Optical steering; triggered release; photothermal therapy.	[43,44,45,46,47,48,49]
	Electric field	Gastric and intestinal mucosa.	Mucosal fixation; hemostasis; delivery; sensing.	[50,51,52]
Chemical Actuation	Hydrogen peroxide	Inflamed intestine and colon.	Inflammation-responsive propulsion; delivery; biofilm disruption.	[53,54,55,56,57]
	Enzyme	Stomach; intestine; inflamed colon.	Chemotaxis; mucus penetration; targeted delivery.	[58,59,60,61]
Biological Actuation	Cell	Inflamed stomach and colon, in hybrid systems.	Cellular relay; inflammation targeting.	[62,63,64,65,66]
	Microbial	Intestine; colon; colorectal tumors.	Tumor targeting; biological delivery; anti-inflammatory therapy.	[67,68,69,70,71]
Hybrid Actuation		Stomach; intestine; colorectal lesions.	Multistage navigation; barrier traversal; theranostic intervention.	[72,73,74,75,76,77]

**Table 2:** Comparison of different structural configurations of microrobots.

Structures	Advantages	Limitations	Application Scenarios	References
Helical	Strong propulsive force; suitability for narrow conduits; effective propulsion in low Reynolds number fluidic environments.	Constrained torque capability; challenging intratissue steering.	Thrombus clearance; targeted drug delivery; dynamic hemodynamic navigation.	[28,81,82,83,84,85,86]
Spherical	Simple structure; ease of mass fabrication; swarm controllability; efficient functional integration.	Boundary sensitivity; reduced efficiency in confined spaces.	Targeted cellular delivery; stem cell delivery; tumor penetration; mucus barrier penetration.	[87,88,89,90,91]
Multilegged	Strong environmental adaptability; high stability; multimodal locomotion capability.	Limited actuation and sensing capabilities; constrained payload capacity.	Targeted delivery; drug transport; complex terrain locomotion.	[92,93,94,95,96,97,98,99]
Tubular	Simple structure; switchable locomotion modes; precise microfluidic manipulation.	Reliance on bubble and fuel actuation.	Targeted delivery; drug transport; complex terrain locomotion.	[100,101]
Star-shaped	Controllable propulsion; favorable biodegradability; excellent mechanical properties.	Limited maneuverability; challenging localization and retrieval.	Directional propulsion; drug delivery.	[102]
Rocket-like	Stable structure; strong propulsive force; effective self-propulsion; controlled drug release.	Reliance on fuel actuation; unstable directional control.	Drug delivery and detection; gastric mucosal penetration; sustained drug release.	[46,103]
Guidewire-like	Large deflection angle; flexible three-dimensional manipulation; precise directional control.	Limited tip steering capability.	Precise navigation; complex luminal traversal.	[104]
Modular	High functionality; modular assembly and disassembly; multimodal locomotion; monolithic integration.	High complexity of cooperative control; trade-off between efficiency and precision.	Lesion detection; transport in high-viscosity environments; obstacle surmounting; cargo handling and retrieval.	[34,80]

## Data Availability

No new data were created or analyzed in this study. Data sharing is not applicable to this article.
